# Supplementary material for: Are carbon emissions trading and green financial instruments synergistic? -Comprehensive quantitative research based on content analysis
Source: PLoS One. 2024 Mar 7;19(3):e0298601. doi: 10.1371/journal.pone.0298601 (PMC10919601; doi:10.1371/journal.pone.0298601)
Supplement: S1 Data — (ZIP) [file pone.0298601.s001.zip › Data/地级市Z_F工作报告.zip!/╔╜╬≈/│ñ╓╬╩╨/└┤╫╘╣1⁄2╓┌║┼í╛┬φ┐╦╩2╛▌═°í┐_2017.docx]

各位代表：

现在，我代表市人民政府向大会报告工作，请予审议，并请市政协委员和其他列席人员提出意见。

一、过去五年工作回顾

过去五年是我市发展极不平凡的五年。我们深入学习贯彻党的十八大、十八届三中、四中、五中、六中全会和习近平总书记系列重要讲话精神，坚决落实省委、省政府一系列部署要求，在市委的坚强领导下，牢固树立新发展理念，稳增长、促改革、调结构、惠民生、防风险，顶住压力、迎难而上，众志成城、砥砺前行，经济社会发展各项工作都取得新成效，在全面建成小康社会征程中迈出坚实步伐。

五年来，我们全力以赴抓项目、帮企业，经济实力有了新提升。不断加强对经济运行的预警预判，宏观层面抓指导，微观层面抓落实，扎实开展项目受理大起底、干部入企服务，着力推进项目建设，逐步形成发展新优势，经济运行持续向好，经济发展平稳健康。一批如高科LED、康宝基因疫苗等科技含量高、带动就业多的好项目建成投产，一批如潞安180、漳泽电力2×100万千瓦“关小上大”等投资大、见效快、支撑能力强的大项目顺利推进，一批如潞宝己内酰胺、大唐风力发电、欧亚神龙湾开发、中技金谷新型建材等新兴产业项目在长治落地生根，一批如中南铁路大通道、长安高速、黎左高速等方便出行、提升运力的重大交通项目投入运营，一批如太焦高铁、长临高速、长治机场改扩建等群众期盼已久的重大基础设施项目正在加紧建设。切实加大帮扶实体经济力度，积极推进“晋材晋用”，举办了两届长治制造展销推介周，全力落实减税降费政策，为企业减轻负担80多亿元。地区生产总值、固定资产投资、工业增加值总量始终保持在全省前两位。

五年来，我们坚定不移抓转型、调结构，产业升级迈出新步伐。有力有序有度推进供给侧结构性改革，全面完成120万吨煤炭去产能任务，煤炭行业告别了30万吨以下的开采时代，焦炭行业进入了5.5米以上大机焦发展阶段，煤化工行业实现多基联产；生物制药走在全国前沿，光伏光电产业从无到有不断壮大，新兴产业投资占工业投资的比重由2011年的43%提高到62.9%，新兴产业增加值占比由2011年的11.8%提高到13.7%。新增设施蔬菜47.1万亩，达到62.8万亩，发展速度连续五年排全省第一；粮食产量持续稳定，粮经作物种植比例进一步优化。农产品加工龙头企业销售收入由2011年的95.3亿元增加到208.7亿元，年均增长16.9%。能交投长子集运站、太行山农产品物流园、新易达物流园建成运营。举办金秋让利系列促销活动，大力发展电子商务，促进消费稳定增长，社会消费品零售总额由2011年的321亿元增加到566.6亿元，年均增长12%。完成了太行山大峡谷旅游资源整合，新增AAAA级旅游景区4个，壶关、武乡、平顺、黎城成为全国创建全域旅游示范县，旅游总收入由2011年的124.8亿元提高到369.1亿元，年均增长24.2%。服务业增加值年均增长6.5%，高于地区生产总值年均增速1.4个百分点。全市三次产业结构由2011年的4.0:66.3:29.7调整为4.7:51.0:44.3。

五年来，我们攻坚克难抓创新、促改革，经济发展增添新动力。持续推进“放管服效”改革，取消中央指定地方实施行政审批事项64项，承接省级下放审批事项56项，下放市级审批事项22项，实行重大项目联审联批，审批时限进一步缩短。不断完善和改进政府管理方式，实行财政资金预拨付制，加快了征地拆迁和市政工程建设速度。深入开展“大众创业、万众创新”，培育6家省级众创空间，新发展私营企业1.8万户，各类市场主体达到15.9万户，新增小巨人企业53户、小升规企业58户。民营经济增加值占GDP的比重达到46.7%，较2011年提高15.3个百分点。在全省率先建成技术交易市场和技术交易信息平台，完成技术交易额87.2亿元。有效发明专利拥有量达到583件。长治商业银行升级更名为长治银行，全市13家农信社中10家成功改制为农

商行，组建了5家村镇银行，3家企业在新三板挂牌，各类金融机构达到158家，较2011年增加48家，各类金融机构存贷款总额达到3318亿元，较2011年增长44%。长治高新区升级为国家级高新区，长治海关正式批准设立。主动融入国家“一带一路”战略，组织企业赴俄罗斯、蒙古等国开展经贸交流。积极推进开发区改革创新、商事制度改革、国企改制、农村土地承包经营权确权颁证、供销社综合改革。全面实施不动产统一登记。完成了新一轮行政机构改革和党政机关公务用车制度改革。

五年来，我们坚持不懈抓城建、夯基础，城乡面貌发生新变化。深入推进上党城镇群建设，以主城区建设为龙头，主城区市政基础设施建设完成投资200亿元，是上一个五年的2.3倍，新建改造市政道路39条，新增市政道路28公里、面积178万平方米，“三环八纵十二横”路网框架基本形成。新敷设各类管道800多公里，新建14座人行过街天桥，改造3座铁路立交桥和130条背街小巷，新增集中供热面积1700万平方米，新增集中供水用户3万户，主城区辐射带动力进一步增强。以大县城、重点镇、中心村建设为节点，城乡基础设施建设完成投资260亿元，集聚能力显著提升。全市常住人口城镇化率由2011年的43.43%提高到51.53%，年均提高1.62个百分点。全面完成城乡人居环境改善“四大工程”各项任务。全市新增公路通车里程689.7公里，总里程达到1.16万公里。开展了深得人心的“五道五治”行动，成效明显。

五年来，我们不遗余力抓治理、强生态，环境治理取得新成效。在主城区全面禁止露天烧烤、禁止燃放烟花爆竹、禁止燃煤，淘汰燃煤锅炉842台，拆除城中村城边村燃煤土小锅炉4728台，对3.54万台燃煤土小锅炉实施洁净焦替代。2500辆公共自行车、500辆纯电动公交车投入运行，大气污染防治成效明显。三类以上水质断面达标率完成省定目标，“4+2”主要污染物减排任务全面完成。大力推进焦化、电力、钢铁、水泥等高耗能企业对标升级改造，淘汰落后产能1395.5万吨，万元GDP综合能耗累计下降18.4%，工业固废利用率达到67.2%。深入推进浊漳河流域生态综合治理工程，完成水土保持生态治理面积135万亩，治理度达到62%，位居全省第一。完成营造林面积190余万亩，森林覆盖率年均提高1个百分点左右，建成区绿化覆盖率达到46.7%，较2011年提高1.8个百分点。

五年来，我们真心实意抓保障、保稳定，民生福祉又有新增进。公共财政用于民生的支出达到963.6亿元，占到全市公共财政支出的80%以上。新增城镇就业22.6万人，转移农村劳动力21.5万人，稳定脱贫16.1万贫困人口。城镇居民人均可支配收入由2011年的18429元提高到28094元，年均增长8.8%；农村居民人均可支配收入由2011年的7177元提高到11863元，年均增长10.6%，城乡居民收入增速持续快于地区生产总值增速，农村居民收入增幅连续7年快于城镇居民收入增幅。探索建立了联盟校办学模式，主城区建成3所公办幼儿园、启动8所义务教育学校建设工程，5个县区通过国家义务教育均衡发展验收，实施了农村义务教育阶段寄宿制学生免费营养餐工程，新建改扩建200所农村幼儿园。城乡居民养老、医疗保险、大病保险实现全覆盖。新农

合人均筹资标准由230元提高到540元。城市低保标准由月均224元提高到488元；农村低保标准由年均1940元提高到3139元。开展了新生儿出生缺陷干预救助健苗工程。为经济困难的高龄与失能老年人及百岁老年人、重度残疾人发放补贴。加强基层公共文化服务，实施全民健身计划。开工建设各类保障性住房9.7万套，14万居民乔迁新居。完成农村危房改造近5万户，15万农民居住条件得到改善。进一步完善安全生产责任体系，创新夜查突查、“两议一报”制度，连续四年实现事故起数和死亡人数“双下降”。深入开展社会风险大防范、矛盾纠纷大排查，不断完善信访处置机制。始终保持打黑除恶高压态势，严厉打击各类违法犯罪活动，深入推进禁毒大会战，社会治安综合治理得到加强，人民安全感满意度持续提高。有效应对洪涝、冰雹等灾害，最大限度降低了财产损失。

五年来，我们毫不懈怠抓作风、创环境，政府自身建设得到新加强。严格执行人大及其常委会的决议决定，积极支持政协履行职能，共办理人大代表建议1093件、政协提案2209件，办复率100%。不断加强与各民主党派、工商联、无党派人士和人民团体的联系，广泛听取各方意见。扎实开展党的群众路线教育实践活动、“三严三实”专题教育和“两学一做”学习教育，始终把纪律和规矩挺在前面，坚定不移推进政府廉政建设，严格落实中央“八项规定”和国务院“约法三章”，持续加大审计监督力度，“三公”经费持续下降。全面完成“六五”普法，法治长治建设步伐加快。启用新的政务服务中心，建立统一的公共资源交易平台，所有行政审批和公共服务事项全部入驻，市直部门权力清单、责任清单全部公布。开通《直通政务厅》，建立“市长县长接听”制度，政风行风持续改进，与群众更贴近、更贴心。

五年来，我市蝉联全国文明城市、国家卫生城市、国家园林城市、全国双拥模范城市，相继荣获了国家森林城市、国家现代农业示范区、国家公共文化服务体系示范区、全国全民健身示范市、国家智慧城市试点市、中国曲艺名城等十多个国家级荣誉称号，长治的影响力和知名度进一步提升。

各位代表，党的十八届六中全会正式明确习近平总书记的核心地位，体现了党和人民的根本利益，对保证党和国家兴旺发达、长治久安，具有十分重大而深远的意义。过去一年，我们在市委的坚强领导下，不断增强政治意识、大局意识、核心意识、看齐意识，认真贯彻省市第十一次党代会精神，按照省委“一个指引、两手硬”重大思路和要求，围绕把长治打造成为山西重要增长极的目标，坚定不移推进供给侧结构性改革，坚定不移实施创新驱动、转型升级战略，经济运行呈现稳步向好的积极态势，全市地区生产总值完成1269.2亿元、增长4.6%，较2015年加快了7.5个百分点；规模以上工业增加值完成583.1亿元、增长2.5%，较2015年加快了12.4个百分点；一般公共预算收入完成98.5亿元、增长2.2%，较2015年加快了31.5个百分点，主要经济指标扭转了负增长的局面，结束了五年来增速下滑的态势，实现了下半年好于上半年，全年好于上年的目标，为今后进一步好转奠定了基础。

各位代表，过去五年取得这样的成绩实属不易，这是省委、省政府亲切关怀、坚强领导的结果，是市委统揽全局、科学决策的结果，是市人大、市政协大力支持、有效监督的结果，是全市广大干部群众同心同德、团结奋斗的结果。在此，我代表市人民政府，向辛勤奋战在各条战线上的干部群众，向各位人大代表、政协委员、离退休老同志，向各民主党派、工商联、无党派和各人民团体，向驻市部队、武警官兵、公安干警，向所有关心支持参与长治改革发展的社会各界朋友，表示崇高的敬意和衷心的感谢！

我们也清醒地认识到，我市经济社会发展中还存在不少困难和问题。一是经济总量不大、发展不足，与全面建成小康社会、打造成为山西重要增长极的要求差距较大，在全面建成小康社会的39项评价指标中，人均GDP只占目标值的68%、农业劳动生产率只占目标值的46.5%,特别是还有个别指标低于全省平均水平，加速发展的任务十分紧迫。二是产业结构不优，“一煤独大”、三产占比不高的问题没有根本性改变，煤炭产业仍然占到工业经济的三分之二，第三产业仅占GDP的44.3%，与“十三五”时期服务业占比稳定在50%以上目标差距较大，推动战略性新兴产业、现代服务业发展任务艰巨。三是创新能力不足，科研经费投入强度不到全国平均水平的一半，319家规模以上工业企业中仅有国家级、省级企业技术中心19家，创新驱动、科技引领亟待加强。四是资源环境约束加剧，大气、水、土壤污染问题突出，雾霾问题日益成为人民群众关注的焦点；万元GDP能耗指标高于小康社会目标值160%，能耗水平居高不下，土地利用效率偏低，资源利用低级粗放。五是城乡发展不均衡，就业增收困难，财政收支矛盾较大，脱贫攻坚难度大，民生社会事业欠账较多，补短板任务十分繁重。六是发展环境不优，行政办事效率不高，诚信体系不完善，法治观念、创新观念、市场观念还不强，不作为、不会为、乱作为等问题仍一定程度存在。对此，我们将始终坚持问题导向、目标导向、任务导向，采取有效措施，切实加以解决，不辱历史使命，不负人民重托。

二、今后五年主要任务

各位代表，今天的长治正处于一个重大历史关头，经济发展进入新常态，改革进入深水区，全面小康进入决胜期；省第十一次党代会吹响了全省创新驱动、转型升级的号角。以市十一次党代会为标志，长治发展翻开了新的一

页，开启了打赢脱贫攻坚战，全面建成小康社会的新征程；今年年初召开的市委十一届二次全会暨经济工作会议，作出了“紧扣‘两个规划’，突出‘一个中心’，坚持‘两个抓手’，强化‘三大支撑’，推动长治转型综改取得突破性进展”的部署，这为今后发展指明了方向，明确了路径。

各级政府部门要深刻领会、牢牢把握，在市委的坚强领导下，要牢固树立新发展理念，不断深化供给侧结构性改革，坚决不要带血的GDP，不要污染环境破坏生态的GDP，不要掺假带水分的GDP，要努力创造有质量有效益真金白银的GDP、绿水青山可持续发展的GDP、老百姓有实实在在获得感和幸福感的GDP，向着更高质量、更有效率、更加公平、更加可持续的方向发展。要牢固树立全新资源观，切实加快转型发展步伐，统筹利用好煤炭、矿产、文化、生态、国际国内两个市场和政府效率、法治环境、社会诚信等各类资源，特别是要用好用活最具革命性、能动性的人力资源，走出长治创新驱动、转型升级新路。要紧扣“两个规划”、突出“一个中心”，按照中原经济区规划和中原城市群规划，主动融入国家战略，把握正确方向，拓展发展新空间，围绕省委“六大工程”，找准切入点、对接点，着力打造“六大工程”长治版，努力构建具有长治鲜明特色的多元化中高端现代产业体系，实现全市经济社会发展战略重点的转移。要坚持“两个抓手”、强化“三大支撑”，一手抓新兴产业培育，一手抓传统产业升级改造，强化改革支撑、创新支撑、开放支撑，把转型深刻融入经济社会发展的方方面面，主动转型，创新转型，深度转型，全面转型，把长治打造成为山西重要增长极。

未来五年，我们要如期完成21.6万贫困人口全面脱贫的任务；我们要实现第一个百年目标，与全国、全省同步全面建成小康社会；我们要为实现第二个百年目标奠定基础。

——转型发展要有明显成效。供给侧结构性改革和转型综改试验区建设取得突破性进展，煤炭产业增加值占工业增加值的比重持续下降，传统产业在工业中的比重控制在60%以内，传统产业竞争力有效增强；先进装备制造、现代煤化工、新能源新材料、节能环保、军民融合、信息技术、现代医药等产业集群初具雏形，分享经济、数字经济、开放型经济快速发展，战略性新兴产业增加值占工业增加值的比重年均提高一个百分点以上；服务业增加值占地区生产总值的比重稳定在50%以上，民营经济占GDP的比重进一步提高。

——创新驱动能力要有明显提升。开发区改革创新取得重大进展，长治国家高新技术产业开发区发展明显壮大，再建一个国家级开发区，每个县建成一个省级开发区，实现增加值占GDP的比重达到25%以上，真正成为转型综改主战场。科技进步对经济增长的贡献率明显提高，以企业为主体的技术创新体系进一步健全，规模以上工业企业中建立研发机构的占比超过15%，研发经费占地区生产总值的比重达到2.5%，每万人口发明专利拥有量达到3.7件。重点领域和关键环节改革取得全方位进展，各方面体制机制更加完善。

——生态环境要有明显改善。能源资源使用效率大幅提高，能耗和水资源消耗、建设用地、碳排放总量得到有效控制，主要污染物减排指标逐年下降，全面完成国家、省下达任务。二级以上天数达标率、水质断面优良率、受污染耕地安全利用率分别达到80%、90%和90%以上。森林覆盖率年均提高0.5个百分点，建成区绿化覆盖率年均提高1个百分点，主体功能区布局和生态安全屏障基本形成。

——人民生活水平要有明显提高。就业、教育、医疗、养老等公共服务体系更加健全，社会保障水平进一步提高。完成行政区划调整，上党城镇群建设取得更大成效，基本形成城乡发展一体化和区域协调发展新格局，全市常住人口城镇化率达到60%以上。现行标准下的贫困人口全部脱贫，贫困县全部摘帽。城乡居民收入增长持续快于GDP，不断缩小城乡收入差距。社会综合治理现代化水平不断提高，安全稳定工作基础更加巩固，平安长治建设扎实推进，人民群众的安全感、获得感、幸福感明显提升。

各位代表，实现上述目标绝非易事，我们有信心、有决心，在市委的坚强领导下，扛起加快长治发展、造福上党人民的历史责任，奋发有为，恪尽职守，努力破解改革发展稳定的各类难题，扎实做好经济社会发展的每一项工作，全力办好事关人民幸福的每一件事情。

三、2017年工作安排

2017年是新一届政府的开局之年，是全面建成小康社会和实施“十三五”规划的关键之年，也是供给侧结构性改革和转型综改的深化之年。今年政府工作的总体要求是：以习近平总书记系列重要讲话精神和治国理政新理念新思想新战略为根本指针，全面贯彻省、市第十一次党代会和十一届二次全会暨经济工作会议精神，按照省委“一个指引、两手硬”的重大思路和要求，牢固树立新发展理念，坚持稳中求进工作总基调，全面实施创新驱动、转型升级战略，坚持以提高发展质量和效益为中心，以全面深化“双改”为主线，以“对表、对标、对接”为要求，全力创优发展环境，保持全市经济稳步向好、社会和谐稳定，为如期实现脱贫攻坚和全面小康奠定坚实基础，以优异成绩迎接党的十九大胜利召开。

主要预期目标是：地区生产总值增长5.5%左右，固定资产投资增长目标根据新的统计口径设置，社会消费品零售总额增长7%左右，一般公共预算收入与上年持平，城乡居民人均可支配收入分别增长6%左右和6%以上，居民消费价格涨幅控制在3%左右，城镇新增就业4.1万人，城镇登记失业率控制在4.2%以内。

约束性指标是：资源节约方面，万元地区生产总值能耗下降3.5%，万元地区生产总值二氧化碳排放量下降3.9%，万元地区生产总值用水量下降2.8%。环境质量方面，全面完成细颗粒物（PM2.5）浓度下降比例，空气质量优良天数比例，劣V类水体比例，二氧化硫、化学需氧量、氨氮、氮氧化物减排幅度等省下达任务。民生改善方面，农村贫困人口脱贫6.8万人，城镇棚户区住房改造11400套。

今年，重点抓好以下工作：

（一）狠抓“三去一降一补”。

“三去一降一补”是深化供给侧结构性改革的五大任务，是破解我市结构性困局，加快转型升级的根本途径，必须全力落实、落到实处，努力取得更大成效。

坚决去产能。运用市场化、法治化方式推进煤炭去产能，今年关停退出太行王家峪煤业、庄底煤业、师庄煤业3座矿井，压减产能165万吨；妥善做好职工安置和债权债务处置工作，确保职工就业有出路、生活有保障。坚持淘汰落后产能与发展先进产能相结合，争取更多煤矿进入先进产能行列，有序释放先进产能。积极推进钢铁、电力、水泥、焦化等行业的去产能工作。

加力去库存。重点抓好房地产去库存和工业品去库存。加大棚户区改造、城中村改造货币化安置力度，货币化安置比例不低于80%，加强房地产用地供应管控，加快保障房分配，有效化解房地产库存。引导企业适应市场需求安排生产，鼓励企业创新营销模式，加大销售衔接力度。建立大中小微企业配套互补合作机制，深入推进“晋材晋用”，办好第三届长治制造展销推介周，不断提高市内企业使用本市产品的比例。力争全市商品房销售面积增长15%以上，主要工业产品产销率达到95%以上。

多措去杠杆。以降低企业负债率为重点，支持企业市场化、法治化债转股，开展股权融资和应收账款融资，引导金融机构帮助企业优化债务结构。建立企业债务重组和不良资产处置协调机制，有效防范企业债务风险。引导企业走向资本市场，力争壶化集团在主板上市，5家企业在新三板挂牌，不断扩大直接融资规模。

积极降成本。全面落实减税降费政策，进一步清理规范中介服务收费，努力减轻企业税费负担。利用应急周转保障资金、信用增进等方式，支持企业多渠道融取低成本资金。将企业直供电范围扩大到40户以上，降低企业用电成本。完善物流配送网络，提高物流效率，降低物流成本。加强企业内部管理，挖潜增效降低管理成本。深化行政审批制度改革，降低制度性交易成本。

强力补短板。针对制约经济社会发展和民生改善的突出问题，加大补短板力度，提高投资的有效性，做实产业转型、创新驱动、基础设施、民生改善的重点项目，产业转型重点项目完成投资584亿元，重大基础设施项目完成投资65亿元，城乡一体化建设完成投资293亿元，民生社会事业完成投资54亿元。

重中之重是加快补齐脱贫攻坚这个短板。深入实施精准扶贫精准脱贫，全面落实“八大工程二十个专项行动”，加大涉农资金统筹整合力度，简化资金拨付程序。坚持产业为要，大力发展特色农业带动2.4万名贫困人口脱贫，发展光伏发电带动3900名贫困人口脱贫，发展乡村旅游带动2400名贫困人口脱贫，发展农村电商帮扶2000名贫困人口创业。坚持就业为重，通过提供岗位、劳务输出和特色培训，帮助8000名贫困人口实现脱贫，吸纳5800名贫困人口参与专业队造林或参加天然林、公益林管护。坚持教育为基，全面落实“雨露计划”和贫困大学生资助，不断加大对贫困人口上学的支持力度。坚持健康为本，落实各项社会保障和社会救助政策，加强对贫困地区的医疗救助服务，有效解决因病致贫。加大金融扶贫力度，创新精准扶贫小额信贷模式，确保完成小额信贷4.6亿元的任务。实施9000人易地扶贫搬迁，完善贫困村的基础设施、公共服务和村容村貌建设。确保完成291个贫困村退出，6.8万名贫困人口脱贫，沁源省级贫困县摘帽的目标任务。

（二）狠抓实体经济振兴。

振兴实体经济是深化供给侧结构性改革的主要任务，要向实体经济发力、聚力，聚焦产业、企业、企业家，做优存量，做大增量，加快构建具有长治鲜明特色的多元化中高端现代产业体系。

发展壮大战略性新兴产业。瞄准发展前沿，发挥现有新兴产业的基础，加快占领行业高端，形成新的优势产业。信息技术产业，实施大数据战略，培育大数据产业，加快智慧长治建设，推进大数据与工业、现代农业、服务业的深度融合。举办长治第三届互联网大会，筹划建设智慧长治云平台，在生产生活生态领域广泛推广大数据应用。加快推进国家北斗数据中心（山西）分中心、联通、移动、电信等数据中心建设。装备制造业，以智能化发展为方向，重点打造新能源汽车、新能源装备、军民融合、节能环保装备等先进装备制造产业集群，加快推进成功集团30万辆新能源汽车、高科LED、易通低温余热发电机组、太重高性能液压产品等项目建设。新能源新材料，推动新能源示范城市建设，重点打造光伏、风力发电、新型建材、镁铝合金等新能源新材料产业集群，争取“国家光伏发电前沿技术依托基地”，扩大光伏、风电等新能源装机规模，加快推进潞宝10万吨己内酰胺聚合切片、中德铝业汽车轻量化等项目建设。现代生物医药，重点打造党参、连翘、黄芪等道地中药材种植深加工现代生物医药产业集群，加快推进康宝血液制品、振东制药抗肿瘤药物、50万亩连翘种植产业化、太行药业中药提取技改等项目建设。

改造升级传统产业。全力创建“老工业城市和资源型城市产业转型升级示范区”，加快推进综合能源产业基地建设，运用新技术、引入新工艺、使用新装备、研发新产品、探索新模式、催生新业态。全面提升煤炭产业、焦化产业的规模化、机械化、信息化水平。加快实施低热值煤发电和漳泽电力2×100万千瓦“关小上大”项目。稳步提升煤层气抽采水平，年内完成长子5亿立方米煤层气开发项目。以发展精细煤化工和现代煤化工为方向，加快推进潞安180、瑞恒60万吨聚氯乙烯、襄矿20万吨乙二醇、潞宝60万吨烯烃等项目建设。做大做优建筑业，扩大钢结构、装配式混凝土和市政构件预制加工能力，努力构建全产业链生产体系，支持企业开拓市内外市场。严格产业技术、规模、环保、能耗、水耗、用地标准，逐步淘汰高耗能、高耗水、高污染、产能过剩的低端产业，改造取缔没有安全保障的高危企业，提高产业准入门槛。

积极推动大众创业、万众创新。引导规上企业自主创新，充分发挥龙头企业双创主体的示范作用，孵化培育一批创新型中小微企业，扶持一批成长性好的创新型企业，全年新增小升规企业12户以上，小巨人企业5户以上，新创办小微企业3300户以上。大力发展新建基地型、闲置厂房改造型、城市楼宇型等创业基地，推动唯美诺中小企业创业基地、钜星众创空间、天空之城创业基地上水平，年内完成长丰、长轴等5个创业基地建设，实现各类创业者“拎包创业”。

大力发展民营经济。保障非公经济与公有制经济一样平等使用生产要素，公平参与市场竞争，同等受到法律保护。扎实开展民营经济政策落地、产业清底、创新提质、服务提升、融资对接行动。实施专精特新企业培育工程，支持民营企业技术中心创新提质，推进民营企业股份制改造。继续办好“长清班”和各项专业培训，促进民营企业家素质提升。

持续加大实体经济帮扶力度。建立完善干部入企服务长效机制，积极帮助企业解决用电、用水、手续办理、品牌创建等方面的困难和问题。引深项目受理大起底，加快项目联审联批，通过联审联批审批项目300个以上。创新要素配置机制，建立完善矿产资源、土地资源市场化配置机制，建立矿权、地权流转市场，用地指标向转型重点项目、向开发区倾斜。对土地、矿产资源占而不用、低效利用的，通过法律和经济手段予以调整优化，努力实现资源的最优配置。

（三）狠抓农业供给侧结构性改革。

以市场需求为导向，以提升农产品质量为着力点，加快构建现代农业产业体系、生产体系、经营体系、服务体系，加快实现农业向提质增效、可持续发展转变。大力发展现代农业。加快国家现代农业示范区创建，围绕增加绿色优质农产品供给，建成6个省级、20个市级绿色高产高效示范片区，建设八大绿色标准化生产示范基地，加快紫团、方兴、沁州黄等现代农业产业园发展，壮大潞宝金和生、浩润食品等30个骨干龙头企业。大力发展“互联网+现代农业”，推进智慧农业发展。加快发展功能农业，推进药食同源产品开发。新培育省级示范专业合作社20家，新发展家庭农牧场50家，培训新型职业农民8000人。新认证“三品一标”农产品50个以上，举办首届国际农合长治周，培育10家农产品出口企业，促进农产品走向国际市场。粮食总产量稳定在14亿公斤以上，农产品加工龙头企业销售收入增长6.5%以上。

强化农业基础建设。整合强农惠农政策，重点支持农业基础设施建设、农业科技创新、农民增收工程。完善农业保险体系，实现种养加产业全覆盖。切实抓好农田水利建设，新开发水浇地1500亩，建设高标准农田6.8万亩，推广增施有机肥20万亩，测土配方施肥35万亩。扩大机耕机播面积，提高秸秆综合处理率，加大农业废弃物回收处理，推广建设农业废弃物资源化综合利用工程。加强重大动植物疫病防控，加快农产品质量安全信用体系和检测监管体系建设。加大农业科技推广力度，健全基层农技推广体系，重点培养100名农业科技创新与推广专家、1000名村级农技推广员、1万名科技示范户。

深入推进农村综合改革。创新农村土地所有权、承包权、经营权“三权”分置办法，健全农村产权流转交易市场。完成农村土地承包经营权确权登记颁证，推广潞城市农村集体资产股份权能改革试点成果，全面推进村集体经济组织清产核资，对经营性资产开展产权制度改革。深入推进资产收益扶贫试点，确保贫困户资产保值增值。大力推进农村水权和农业综合水价改革。加快推进国有林场改革、集体林权制度改革、农村供销合作社综合改革。

（四）狠抓现代服务业发展。

现代服务业是中高端产业体系重要组成部分，是地区综合竞争力和现代化水平的重要标志。我们要不断拓展现代服务业发展空间，提升现代服务业发展质量和水平，力争服务业占GDP比重提高1个百分点以上。

加快现代物流业发展。以太行山农产品物流园、能交投物流园、紫坊农贸物流园等为龙头，重点发展能源矿产品物流、大宗商品物流、城市配送物流和农产品物流。以智慧物流、多式联运、连锁物流、城乡快递、冷链物流为重点，加快物流基础设施建设，培育现代物流企业集团，延伸物流配送网络。引导商贸物流企业运用电子商务拓展销售规模，培育5至10家市级电商示范企业，新增2个电商孵化基地、500个村级电商服务站。

加快高新技术服务业发展。大力发展研发设计、知识产权、检验检测、科技成果转化、信息技术等高新技术服务业。加快发展以会计、法律、审计、评估、专利代理为重点的咨询服务业，不断推动在重大决策、重大项目和工程技术等领域引入咨询服务。积极发展会展业，培育会展市场主体，延伸会展产业链。

加快传统服务业优化提升。适应大众消费多样化、个性化、信息化需求，推动商贸、餐饮、住宿、家政等行业加快业态创新、管理创新和服务创新，促进线上线下互动发展。开展系列促销活动，推进大型商业综合体建设，打造3条特色餐饮街区，提升商贸服务水平。大力发展健康养老、社区服务、文化休闲等幸福产业。

加快文化旅游业发展。做大做强“太行山水·红色经典”文化旅游品牌，推进神龙湾、太行红山、太行欢乐谷等重点旅游项目建设，力争太行山大峡谷创建成5A级景区。上半年完成18个旅游景区的改制工作。开工建设31项、530公里旅游公路，增设旅游景区标识标牌，新建改建旅游厕所76座。加大旅游宣传推介力度，举办太行经典·长治久安旅游摄影大赛。在壶关、武乡、平顺、黎城4个县，成立旅游发展委员会，探索建立旅游警察、旅游巡回法庭、旅游工商分局，推动全市全域旅游发展。实施“旅游+”，建设全市智慧旅游平台，推动旅游与文化、体育、工业、农业等相关产业融合。继续做好旅游惠民“一卡通”工作，增加旅游专线客车线路。集中开展旅游专项整治行动，优化旅游消费环境。确保全市旅游总收入增长12%以上。

（五）狠抓重点领域改革。

改革是发展的必由之路，解决当前经济社会发展的突出矛盾和问题，根本途径在于深化改革。我们必须攻坚克难、全力突破，推进各项改革取得实实在在的成效。

加速开发区改革创新。按照开发区整合改制扩区调规的要求，合理确定规模，坚持“五规合一”，坚决不碰生态保护区的底线，全部调出起步区内基本农田，加快起步区“九通一平”、标准化厂房建设。统筹产业布局，完成长治高新区的扩区工作，新设立长治经济技术开发区，今后凡是新上项目原则上全部入驻开发区，现有具备条件的项目逐步向开发区搬迁集聚，开发区内原有企业同主导产业不相符的逐步“腾笼换鸟”。理顺开发区管理体制、运行机制，今年5月份前完成开发区“三化三制三改”工作，强化开发区经济属性，吸引更多大企业、大集团落户。确保襄垣、潞城两个工业园区升级为省级经济技术开发区，努力使开发区成为全市转型综改的主战场。

加速国资国企改革。对恢复生产无望的“僵尸企业”、“空壳企业”和问题突出的国有集体企业，创造条件推动企业采取兼并重组、产权划转、破产关闭等方式进行改制，妥善安置职工，解决历史遗留问题，有效盘活国有企业闲置资产和土地，年内完成20户企业的改制工作。全面完成行政机关与所办企业脱钩改革任务，建立完善国有资产集中统一监管平台，切实加强国有资产监督管理，不断提高国有资本收益。

加速投资体制改革。全面对标发达地区投资环境，大力推进投资审批改革，全面推行网上审批，坚持多评合一、多审合一、多图联审、联合验收，对技术审查类审批实行先建后验模式，积极探索开展企业投资项目承诺制试点。主动争取上级各类专项资金，积极引进世行、亚行、亚投行等国际资金。大力推广政府与社会资本合作的PPP模式，力争今年有50个项目落地实施。按照财政政策金融化、财政资金杠杆化的思路，发挥民营经济转型发展基金、扶贫开发基金、美丽乡村建设基金等政府性基金的引导作用，利用城投公司、财鑫担保公司等平台，支持重大改革、重大项目、重点工程建设。

加速金融改革创新。促进长治银行做优做强做大，支持设立村镇银行，加快推进黎城、沁县、沁源的信用社改制步伐，力争在全省率先实现农商行全覆盖。完善政银企联席会议制度，加强政银企对接，提升信贷支持实体经济的质量和效益。推动民间融资综合服务平台、股权登记托管平台、应收账款债权流转平台、金融信息服务平台实质性运作。促进互联网金融健康发展。用好保险资金防灾防损功能，发挥保险资金投融资功能，推广贷款保证保险，探索吸收保险资金支持经济建设。

（六）狠抓科技人才质量强市战略。科技是第一生产力，人才是第一要素，质量是第一保障。我们必须在强化科技引领、发挥人才作用、提升质量水平上下实功、出实招、求实效。

全面推进科技强市。积极争取国家创新型城市试点，强化企业创新主体地位，支持企业深化产学研合作，新增2个省级技术中心、5户国家高新技术企业。加快推进技术成果和知识产权交易，推进技术交易额持续增长，促进科技成果在我市落地转化。筹建长治市工业技术研究院，实施20项以上科技重点攻关项目。建立科技资源共建共享机制，促进大型科学仪器和科技资源共用共享。进一步加大知识产权保护力度，有效发明专利拥有量达到600件以上。

全面推进人才强市。创新“产业资本+人力资本”模式，实施“潞商潞才回乡创业创新工程”，力争引进2至3家企业的核心研发团队。创新用人机制，实行以增加知识价值为导向的分配政策。统筹推进各类人才特别是实用人才和高技能人才的培养，鼓励小发明、小创造、小制作，弘扬工匠精神，培育一批精益求精独具特色的长治工匠。

全面推进质量强市。牢固树立质量第一、品牌为王、标准先行的理念。深入开展质量提升行动，不断提高产品质量，增强产品竞争力，以质量抓提升、占市场、促转型。开展品牌创建行动，积极参加山西品牌“中华行”“丝路行”“网上行”，打造潞商品牌，今年力争推出10家山西省名牌企业，2个地理标志保护产品。加快质量标准化体系建设，支持企业参与国家标准制定，选树15家以上质量提升标杆企业，力争制定3至5个新兴产业产品的行业标准，推动更多的企业标准、地方标准上升为国家标准。全面开工建设国家煤基油质检中心。实施计量惠民工程，推动社会诚信体系建设，争创全国质量强市示范城市。

（七）狠抓开放型经济发展。

提升开放型经济发展水平是内陆地区实现追赶发展的关键所在。我们必须全力推进全方位、高质量的对外开放，以开放促改革、以开放促发展。

全面落实“东融南承西联北拓”战略。主动参与中原城市群建设，强化与中原经济区的对接联系。积极融入环渤海经济圈，深化与首钢集团的全方位合作。加快融入国家“一带一路”发展战略，组织企业赴沿线国家开展经贸交流活动，拓展经贸合作领域。积极承接长三角、珠三角、港澳台地区的产业梯度转移。不断扩大太阳能电池片、硝酸铵、新型建材、农产品等优势产品出口规模，新培育3至5家出口创汇企业，全年出口额增长3%以上。

加快推进基础设施内通外联。不断完善综合交通网络，优化提升“铁、公、机”。加快太焦客专、长治机场改扩建、长临高速、长邯高速改扩建等项目建设，争取早日建成投入运行，积极争取黎霍高速公路开工建设，畅通对外联系通道。加快建设“岸、港、网”，在中南铁路长子南编组站设立铁路口岸作业区，推进无水港建设，力争长治机场航空口岸实现临时开放。进一步完善互联网、物联网等信息网络设施，推动“互联网＋”工程实施。抓好海关综合服务设施和出入境检验检疫技术业务用房建设，力争年内主体完工。

更加注重招商引资成效。把开发区作为招商引资的重要平台，围绕产业链条、产业集群、产业目录，创新招商方式，充实招商引资项目库、客商库、信息库、人才库，编制“招商地图”，实施精准招商和跟踪服务，促进项目落地投产。充分发挥驻外办事处在招商引资中的窗口和桥梁作用，组织开展专业性、系统性专场推介对接活动。加大对签约项目的开工率、资金到位率进行考核，力争境外到位资金达到5亿美元以上。

（八）狠抓城乡一体化发展。

城乡一体化是推进新型城镇化的必由之路。我们必须坚持以“五个统筹”为引领，以“五规合一”为路径，以城中村、棚户区改造为主线，优化城乡空间布局，加快上党城镇群建设，推动城乡共同繁荣。

推进主城区扩容提质。全力推动行政区划调整，优化城市发展框架，全面启动主城区城中村、棚户区改造，年内完成36个城中村整村拆除任务，新开工棚户区改造11400套，基本建成8000套。围绕行政区划调整、城中村改造、棚户区改造，开工建设一批市政道路、桥梁等市政基础设施工程。启动机场与高铁站、东客站的快速通道建设。积极推进海绵城市建设，加大黑臭水体治理力度。推进污水处理、污泥处置、生活垃圾发电等PPP项目建设。加快太行公园改造和人民公园动物区搬迁。全力推进207、208、309国道过境段改线工程，全面完成“三河一渠”综合治理，形成主城区20公里长的环城水系、112公里的环城公路、135公里的绕城高速的三环格局。

提升城乡综合管理水平。引深“五道五治”行动，形成常抓常管严抓严管的环境整治长效机制，从根本上改善城乡人居环境。深入推进城市执法体制改革，实现市县政府城市管理机构的综合设置，行政处罚权的集中行使。实施路灯节能改造和街道亮化提升工程，鼓励有条件的既有建筑增设电梯。加快智慧城市建设，促进大数据、物联网、云计算等现代信息技术与城市管理服务深度融合，提升智能化管理水平。做好国家卫生城市的复审验收工作。

加快上党城镇群建设。推动主城区与周边县市的设施对接、产业对接、机制对接、服务对接，加快推进规划编制、基础设施、公共服务、产业布局、制度安排“五个一体化”，规划建设一批特色小镇，积极推进医疗、教育等基本公共服务城乡均等化，有序推动水电路气等基础设施城乡联网、共建共享。促进符合条件的农业转移人口有序落户城镇，稳步推进城镇基本公共服务常住人口全覆盖，使农业转移人口与城镇居民享有同等权利和义务。

建设美丽宜居乡村。大力实施农村人居环境改善四大工程。以贫困村脱贫、沉陷村搬迁、城中村改造、中心村提升、文化村发展旅游为重点，抓好“改气、改厕、改水、改电”，切实改善农村生产生活条件。新创建12个省级、20个市级美丽宜居示范村，改造农村危房7038户，推进5个农村垃圾治理试点县建设，所有行政村全部建成乡村清洁工程达标村。

（九）狠抓文化软实力培育。

上党文化源远流长，底蕴深厚，发展潜力巨大。我们要像挖掘地下资源一样挖掘文化资源，像抓经济建设一样抓文化建设。

完善公共文化服务体系。巩固提升国家公共文化服务体系示范区创建成果，加快推进市博物馆、图书馆、档案馆建设。完善市县乡村公共文化服务设施，实施数字图书馆、电子阅览室、数字农家书屋等公共文化数字服务项目。实施优秀传统文化传承发展工程和乡村文化记忆工程。启动文化资源大调查，动员社会力量参与文物保护利用，深入挖掘、保护、传承非物质文化遗产。大力推进文化惠民。继续办好周末大剧院、流动图书车、农村公益电影放映、上党文化大讲堂等文化惠民活动。倡导全民阅读，建设书香社会。加强网络文化建设，净化网络环境。做好修志编鉴工作。强化文化艺术人才队伍建设和文化市场管理，加快推进市梆子剧团、落子剧团、豫剧团、杂技团四个院团的排练场地建设，力争年内投入使用。

加快发展文化产业。做好文化产业统计普查。促进文化与旅游、科技、信息、体育、金融等产业融合发展。推动传统媒体与新兴媒体深度融合。办好武乡八路军文化旅游节、黎城太行红山国际自行车骑游文化节、沁县端午民俗文化节等文化节庆活动。积极发展文化创意、动漫游戏、数字出版等新业态，支持中小微等各层次文化企业发展，着力打造一批市场化的产业基地。

加强精神文明建设。牢固树立社会主义核心价值观，大力弘扬太行精神、纪兰精神，深入开展社会公德、职业道德、家庭美德和个人品德教育，选树长治好人、道德模范、文明家庭，培育良好家风。推动移风易俗，树立文明乡风民风。拓展群众性精神文明创建活动，争创第五届全国文明城市，提高全社会文明水平。

（十）狠抓生态文明建设。

绿水青山就是金山银山。生态环境没有替代品，用之不觉、失之难存。我们要顺应人民群众关切，坚持不懈加强生态文明建设，努力建设山清水秀、天蓝地绿的美丽家园。

突出大气污染防治。严格落实京津冀大气污染“2+26”城市群联防联控，把全市划分为大气污染防治敏感区、严控区、监管区，实施按区预警、因区施策。不断深化“控煤、治污、管车、降尘”措施，全面开展县城建成区燃煤锅炉整治，实施主城区及周边农村冬季取暖煤改电、煤改气替代工程。开展“小散乱污”企业专项取缔行动，实施工业企业冬季采暖期错峰生产。持续提升油品质量，推进机动车尾气污染防治。强化扬尘污染控制。实施20项节能技术改造项目，加强企业节能降耗。持续保持环境执法高压态势，对偷排偷放、数据造假、屡查屡犯的企业依法严肃查处、上限处罚。

强化水污染防治。全面实施“河长制”，综合利用好地表水，严控地下水开采。全力保护上党盆地、长治湿地，完成5项湿地生态工程。辛安泉供水和东山供水工程正式投用，加快推进县域小水网建设。全面完成县城污水处理厂提标改造，加快工业集聚区污水处理厂建设，实现城镇生活污水、工业园区污水直排清零，确保水质断面达标。

注重土壤污染防治。落实最严格的耕地保护制度，牢守539万亩耕地红线。全面落实国家《土壤污染防治行动计划》，启动农用地污染状况详查。加大土壤环境监测力度，实施农用地分类管理，加强农业面源污染防治，实施化肥农药零增长行动，保障农业生产环境安全。开展污染场地环境风险评估制度建设，明确保护、禁止、限制的措施要求。

创新环境保护机制。启动生态保护红线划定，编制《长治市生态保护红线划定方案》，合理确定县市区生态保护红线划定的区域和面积。坚持资源集约节约高效利用，把土地产出率作为重要考核指标，提高土地集约节约高效利用水平。大力推行排污者付全费的环境污染第三方治理，全面实施主要污染物排污权有偿使用和交易制度。扩大企业在线监测范围，重点企业监测、重大项目验收全覆盖，强化环境风险防控，提升预警、应急处置能力，确保全市环境安全。

打造生态安全屏障。加强以太行山绿化、退耕还林为重点的林业生态建设，推进林业产业提质增效，强化森林资源管护，完成营造林面积30万亩。创建1个森林县城、10个森林乡镇、100个森林村庄。抓好浊漳河南源河流生态修复工程，治理水土流失面积45万亩，完成2566户的采煤沉陷区治理。

（十一）狠抓民生改善。

民生是为政之要，必须时刻放在心头、扛在肩上，要持之以恒为群众办实事、解难事，促进社会公平正义，增进人民福祉。

扎实办好“六件实事”。今年省政府确定了要为广大群众办好的“六件实事”，我们要全力落实、抓紧抓好。一是实施4410名残疾预防重点干预和残疾儿童抢救性康复项目。二是对3个国家贫困县3.2万名建档立卡的农村妇女免费进行“两癌”筛查。三是为全市城乡怀孕妇女提供免费产前检查和诊断服务。四是新建79个农村老年人日间照料中心。五是实施8000名建档立卡农村贫困劳动力免费职业培训。六是免费送戏下乡800场。

着力促进就业创业。全面落实创业就业扶持政策，实施职业培训全覆盖计划，不断深化创业型城市建设。推进高校毕业生就业创业促进计划，实施“三支一扶”、就业见习等基层服务项目，吸纳更多高校毕业生就业。扎实开展就业援助专项行动，托底帮扶就业困难人员，确保零就业家庭动态清零。做好城镇失业人员、“4050”人群、退役军人、残疾人等群体就业工作。培育农村劳务经纪人，提高农村劳务输出组织化程度。

优先发展教育事业。认定扶持90所普惠性幼儿园，确保学前三年毛入园率达到90%以上，全面规范学前教育办学。主城区8所义务教育学校建设主体基本完工，完成180所农村寄宿制学校的标准化建设，确保长子县、长治县今年通过国家义务教育发展基本均衡县验收。主动适应高考改革，全市所有普通高中办学条件基本通过达标验收，稳定提升高中阶段教育水平。科学设置专业，加强产教融合，开工建设职教园区，加快推进体校搬迁，推进职业教育健康发展。落实“1331”工程，加大市校合作，加快发展高等教育，长治幼儿师范高等专科学校力争秋季先期招生。加强师德师风建设，不断提升教学研究水平。

完善社会保障体系。积极推进养老保险制度改革和城乡居民医保制度整合。提高城乡低保标准，推动脱贫线、低保线有效衔接。提高城乡基本医保财政补助标准。不断完善社会救助体系，加强流浪乞讨人员和流浪未成年人救助。推进殡仪基础设施建设，加快殡葬业改革。加快推进城市社区养老中心建设，实施残疾人精准康复服务行动。锲而不舍解决农民工工资拖欠问题，决不允许他们的辛勤付出得不到应有的回报。全面实施全民参保登记，逐步消除社会保障覆盖盲点，实现人人享有基本社会保障。

加快发展健康事业。加快市医院、市二院综合楼、市妇幼保健院改扩建、市中医院、精神卫生中心、突发公共卫生事件救治基地、市儿童医院等项目建设。落实医药流通领域“两票制”改革，推进城乡药品配送一体化。积极推进县乡医疗卫生机构一体化改革，提升市县医疗机构服务能力。实施乡村卫生标准化覆盖和健康扶贫工程，提高乡村医生待遇。全面落实两孩政策，继续实施新生儿疾病筛查“健苗工程”。加强公共卫生安全体系建设，争创食品安全城市，确保食品药品安全。推进全民健身和全民健康深度融合，积极争取、主动创办大型赛事活动，做好第二届全国青年运动会长治赛区的筹备工作。

发展妇女儿童、老龄、慈善和红十字会等事业，加强气象、地震、人防、科普、档案、双拥共建、地方志等工作。做好民族宗教、外事、侨务、港澳、对台等工作。扎实做好国防教育、国防动员、民兵预备役工作。继续做好对口援疆工作。

（十二）狠抓安全稳定工作。

人命关天、安全至上。我们一定要牢固树立总体安全观，以铁的担当尽责，铁的手腕治患，铁的心肠问责，铁的办法治本，坚决守住“三条底线”，绝不让安全稳定问题影响干扰转型发展大局。

压实安全生产责任。坚持“党政同责、一岗双责、企业主责、失职追责”，按照“管行业必须管安全、管业务必须管安全、管生产经营必须管安全”的要求，严格落实各级各部门的安全生产监管责任。全面推行安全生产挂牌责任制，实现所有工商登记注册生产经营单位安全监管全覆盖。严格落实企业安全生产主体责任，切实做到安全责任、管理、投入、培训和应急救援“五到位”。强化安全生产监管。健全安全生产夜查突查和“两议一报”制度，始终保持安全监管高压态势。进一步优化安委会组织架构，设立多个行业专委会，构建运行高效的安全生产协调机制和组织管理格局。充实市县两级安全生产监管执法人员，强化乡镇（街道）监管力量，完善开发区、工业园区、风景区等功能区的安全生产监管。抓好山西省应急救援长治基地建设，运用现代化手段加强监管，切实提高应急处置能力。

强力整治安全隐患。全面开展安全风险排查和评估，从人防、物防、技防等方面制定管控措施，实行差异化动态监管。在各行业各领域全面开展安全隐患大排查大整治，严厉打击各类非法违法生产经营建设行为，真正把隐患当事故对待，坚持问题导向，对隐患问题盯死盯牢，对查处的隐患不解决不放过，实现隐患动态清零，确保安全生产事故起数、死亡人数持续“双下降”，全市安全生产形势保持稳定。

维护社会大局稳定。深化社区“网格化”管理，加强对特殊人群的帮扶和管理，做好社区矫正工作。创造良好金融生态环境，严厉打击恶意逃废金融债务和非法集资活动。扎实做好信访工作，完善社会稳定风险评估机制，有效调处化解各领域矛盾纠纷。健全应急管理和防灾减灾体系，不断提高突发事件应急处置能力。深入推进平安长治建设，持续打好“六大战役”，狠抓社会治安综合治理，努力营造和谐稳定的社会环境。

四、切实加强政府自身建设

推动转型发展，创优发展环境，政府肩负着最直接、最重要的责任，我们要义无反顾地把如期实现脱贫攻坚、全面建成小康社会这一历史重任担当起来，始终坚持党的领导，始终尽心履职尽责，始终忠诚干净担当，切实加强人民政府、法治政府、效能政府、担当政府、廉洁政府建设，不断提升政府治理体系和治理能力现代化水平。

始终坚持为民尽责。牢固树立以人为中心的发展思想，全心全意为人民服务是我们的根本宗旨，所有工作都要体现人民意愿、维护人民利益、接受人民监督。全面推行行政决策、执行、管理、服务、结果公开，保障人民群众的知情权、参与权、表达权、监督权。坚决执行人大及其常委会决议决定，依法向人大及其常委会报告工作，主动接受人大法律监督、工作监督、政协民主监督、人民群众监督和社会舆论监督，认真办理人大代表建议、政协提案、人民群众来信来访，件件有回音、事事有着落。加强与各民主党派、工商联、无党派人士和人民团体联系，积极支持工会、共青团、妇联等人民团体开展工作，广泛听取各方面意见建议。畅通与群众沟通渠道，坚持群众路线，重大决策问计于民、问需于民，主动深入基层、深入一线，真正帮助群众解决实际困难和问题。

始终坚持依法行政。深入贯彻全面依法治国要求，尊崇法治、敬畏法律、依法办事。坚持科学决策、民主决策、依法决策，实行重大行政决策合法性审查机制、终身责任追究制和责任倒查机制。加大决策咨询力度，推进政府智库建设。全面推行政府法律顾问制度，加强行政复议和应诉工作，扎实做好地方立法工作。积极推进综合行政执法体制改革，探索建立行政执法全过程记录、重大行政执法决定法制审查等制度。全面推开“双随机、一公开”，随机确定执法人员和检查对象，公开执法结果。深入开展“七五”普法，加强公共法律服务体系建设，搞好法律援助工作。

始终坚持创优环境。按照审批最少、流程最优、体制最顺、机制最活、效率最高、服务最好的要求，扎实推进“放管服效”改革，推进“互联网+政务服务”，进一步完善项目联审联批制度，推行投资审批网上办理，让企业、群众办事更高效、更便捷。持续推进商事制度改革，切实加强事中事后监管。建立覆盖市县乡政府的“13710”电子督办平台，构建起横向到边、纵向到底的抓落实体系，将重大决策部署、重要改革事项、重点工作任务、重点产业项目纳入平台管理实行限时办结，见人见事、见根见果、确保实效。畅通“直通政务厅”，办好“市长县长接听”，做到企业问题有处说，群众问题有人答。始终坚持干事担当。我们要干字当头、真抓实干、埋头苦干。改进政府工作方式，扎实推进基层组织、基础工作、基本能力建设，强化政策导向，细化行动目标，精准方案要求，实行重点工作清单化、项目化、责任化管理，建立清晰明确的责任可追溯体系。面对艰难险阻、不推不躲，知难而进、迎难而上，以逢山开路、遇水架桥的勇气，在全面深化改革中攻坚克难，在履职尽责中推动发展，切实把责任扛起来，把担子挑起来，真正做到守土有责、守土负责、守土尽责。建立健全激励机制和容错纠错机制，鼓励大胆创新，宽容合理失误，对工作主动作为、行动迅速、成效明显的予以褒奖，对工作落实不力、效能低下、敷衍塞责、弄虚作假的慢作为、不作为、假落实、不落实行为严肃追责问责。

始终坚持廉洁自律。认真贯彻党的十八届六中全会精神，严格执行《准则》《条例》，履行好各级政府党组落实全面从严治党任务的主体责任，严肃党内政治生活，深入推进政府系统党风廉政建设和反腐败斗争。坚决把纪律和规矩挺在前面，健全“把权力关进制度笼子”的长效机制。加强审计监督，强化对重点领域、关键环节的监督制约。牢固树立过紧日子思想，拒奢尚俭，严控“三公”经费支出。严格落实中央“八项规定”和国务院“约法三章”，一以贯之纠正“四风”，严查损害群众利益的不正之风和腐败问题。全体政府公务人员要始终持廉守正，干干净净为人民做事。

各位代表，转型发展时不我待，全面小康只争朝夕。让我们在省委、省政府和市委的坚强领导下，团结带领全市人民，凝心聚力，开拓进取，撸起袖子加油干，努力把长治打造成为山西重要增长极，为如期实现脱贫攻坚和全面建成小康社会目标而努力奋斗，以优异成绩迎接党的十九大胜利召开！
